# Performance of Flexible Chemoresistive Gas Sensors after Having Undergone Automated Bending Tests †

**DOI:** 10.3390/s19235190

**Published:** 2019-11-27

**Authors:** Miriam Alvarado, Silvia De La Flor, Eduard Llobet, Alfonso Romero, José Luis Ramírez

**Affiliations:** 1 Departament d’Enginyeria Electrònica, Elèctrica i Automàtica, ETSE, Universitat Rovira i Virgili, Av. Països Catalans 26, 43007 Tarragona, Spain; miriam.alvarado@urv.cat (M.A.); eduard.llobet@urv.cat (E.L.); alfonso.romero@urv.cat (A.R.); 2 Departament d’Enginyeria Mecànica, ETSEQ, Universitat Rovira i Virgili, Av. Països Catalans 26, 43007 Tarragona, Spain; silvia.delaflor@urv.cat

**Keywords:** gas sensor, flexible sensor, metal oxide, nanowires, polymeric substrate, bending tests

## Abstract

Many sensors are developed over flexible substrates to be used as wearables, which does not guarantee that they will actually withstand being bent. This work evaluates the gas sensing performance of metal oxide devices of three different types, before and after having undergone automated, repetitive bending tests. These tests were aimed at demonstrating that the fabricated sensors were actually flexible, which cannot be taken for granted beforehand. The active layer in these sensors consisted of WO_3_ nanowires (NWs) grown directly over a Kapton foil by means of the aerosol-assisted chemical vapor deposition. Their response to different H_2_ concentrations was measured at first. Then, they were cyclically bent, and finally, their response to H_2_ was measured again. Sensors based on pristine WO_3_-NWs over Ag electrodes and on Pd-decorated NWs over Au electrodes maintained their performance after having been bent. Ag electrodes covered with Pd-decorated NWs became fragile and lost their usefulness. To summarize, two different types of truly flexible metal oxide gas sensor were fabricated, whereas a third one was not flexible, despite being grown over a flexible substrate following the same method. Finally, we recommend that one standard bending test procedure should be established to clearly determine the flexibility of a sensor considering its intended application.

## 1. Introduction

Nowadays, great efforts are being made to develop different types of flexible electronic devices, such as diodes, transistors, displays, and sensors. One main application for these flexible devices is to work attached to the human body, or integrated in fabrics and clothes, becoming what is known as “*wearables*”. Sensors are among the most developed devices for wearable applications. Wearable sensors are used to monitor a wide range of variables such as physical activity, heart rate, or respiratory frequency. Most of the currently marketed wearables are aimed at monitoring training/fitness activities. However, this is evolving rapidly to application fields such as health, environmental security, food quality, and many more. Frequently, these applications require the development of gas sensors that should be flexible if used as wearables.

According to the sensing principle, we can find electrochemical, chemoresistive, piezoresistive/piezoelectric, and Hall Effect gas sensors that are both rigid and flexible, among others [1,2,3,4,5,6]. Chemoresistive sensors are based on materials that change their electrical conductivity in response to an interaction with their chemical environment [7]. 

The development of flexible chemoresistive gas sensors implies a big challenge, because most of the gas sensitive materials currently used in chemoresistors (e.g., carbon nanotubes, conducting polymers, metal oxides, graphene, and metals) require high synthesis and operating temperatures. Furthermore, the vast majority of flexible substrates (e.g., plastic, paper, and textile) do not withstand high temperatures. Therefore, it becomes essential to harmonize the properties of the flexible substrates with those of sensing materials and associated fabrication methods. Moreover, truly flexible sensors must cope with repeated mechanical stresses (i.e., bending) throughout their lifetime without suffering important changes in their sensing properties. That is, not only must the substrate be flexible, but the sensor must also continue operating as such after it has been bent. 

One of the most used one-dimensional (1D) nanostructures for the manufacture of flexible electronic devices is nanowires [8]. Metal oxide nanowires are ideal for gas sensor fabrication owing to their high surface-to-volume ratio, their superior stability, the possibility of reaching high integration densities, and their easy integration into microelectronic devices [9]. Recently, nanowires have been used to develop a variety of gas sensors employing flexible substrates [10,11,12,13]. Despite the remarkable results achieved, their flexibility and response under cyclic bending have been barely studied. In addition, there is neither a consensus nor a standardization defined in the existing literature about what procedures or tests should be implemented for assessing the flexibility of gas sensors. A standard test methodology would allow an adequate comparison between the results reported. Generally, the parameters used for describing bending tests are the angle, radius, and number of cycles over which the bending test is repeated. However, these parameters are not specific enough to enable a completely meaningful comparison between the results reported by different authors. On the one hand, bending “speed” and cadence are usually missing, despite being essential parameters to value the mechanical stress applied. On the other hand, even bending angles are not necessarily comparable if the distance between gripping points varies or additional supports are used to prevent substrates from freely flexing over defect structures. In addition, some flexible sensors are reported in which bending tests are totally missing. Table A1 and the references herein summarize the results of the flexural tests applied on allegedly flexible gas sensors, in which metal oxide (MOX) nanowires/nanorods (NWs/NRs) were used as the sensitive material.

This paper shows a procedure applied to three different types of MOX gas sensors in order to compare their performance before and after being bent. Firstly, sensor response against H_2_ was measured and recorded. Secondly, sensors were subjected to automated bending tests using specialized equipment, which allowed precisely controlling and repeating the applied stress. Lastly, the measurements of sensor response to different concentrations of H_2_ were repeated. All three types of sensors were fabricated growing WO_3_ NWs on top of a polyimide foil by means of the aerosol-assisted chemical vapor deposition (AA-CVD). Despite sharing most of the materials and methods, two of the sensor types showed very good performance after the bending test, while one became fragile and lost integrity after a few cycles. Thus, the results demonstrated, on the one hand, the suitability of the proposed procedure to fabricate affordable flexible gas sensors, and on the other hand, that not every sensor fabricated using a flexible substrate is able to endure being actually flexed.

## 2. Materials and Methods

### 2.1. Design and Materials

Three different types of sensors were fabricated. All of them shared the same substrate material and electrical design: a flexible polymeric substrate, a metallic electrode, and a metallic coplanar heater printed on top of the substrate. The electrode, heater, and the gap between them constituted the sensor active area. The active area was coated with a WO_3_ NW sensing layer. Since the layout had only one electrode, the heater acted both as a second electrode and a heating element simultaneously, following the same approach used in [14]. A piece of Kapton^®^ HPP-ST (50.8 µm thickness) was used as the flexible polymeric substrate. The difference between each type of sensor came from the metallic ink used to print the electrode/heater and whether or not the WO_3_ NWs were decorated with palladium nanoparticles.

#### 2.1.1. Silver Stenciled Transducers with Pristine WO_3_ NWs Active Layer

**Figure 1:**
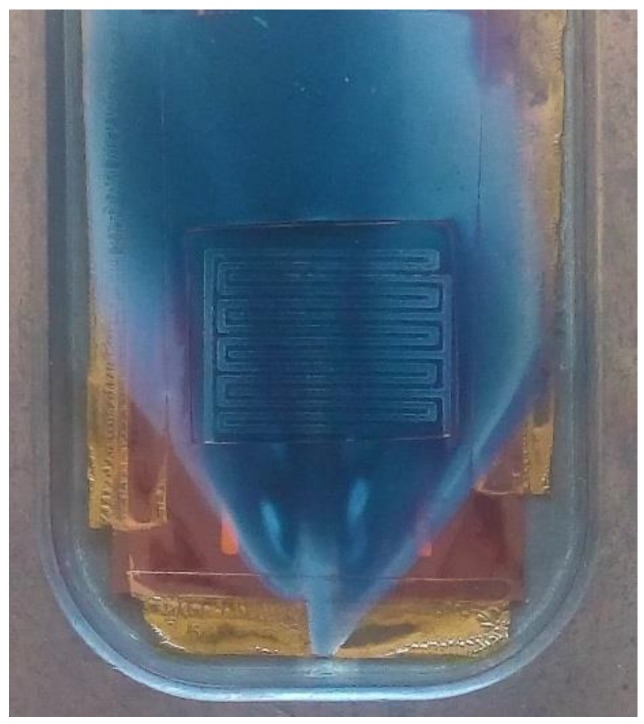
View of the steel hot wall reactor, opened after the aerosol-assisted chemical vapor deposition (AA-CVD) process. Kapton mask (brown), Ag stenciled tracks, and grown WO_3_ (blue) can be observed.

Silver stenciled transducers were designed with a size of 19.30 mm × 15.55 mm, and a sensing area of 12.50 mm × 15.55 mm. The track width and gap between the electrodes were 400 µm. Electrode and heater patterns were stenciled using silver ink (DuPont^®^ 5064H, silver conductor, Wilmington, DE, USA). Firstly, substrates were cleaned in an acetone bath for 5 min, followed by an ethanol bath for 5 min and, at last, rinsed with deionized water and dried at 110 °C for 10 min. Once substrates were cleaned, the stencil technique used in [15] was employed to deposit the electrode and heater onto the substrate. The WO_3_ NWs active layer was grown on the active area of the flexible substrates via the AA-CVD method using a hot wall reactor. A mask of Kapton^®^ was used to define the area of the active layer, avoiding the deposition of highly resistive oxide on top of the contacts (Figure 1). The active layer was grown at 350 °C. For all the depositions, the reactor was heated at a rate of 1 °C per min. To obtain WO_3_ pristine nanowires, 50 mg of tungsten hexacarbonyl (W(CO)_6_, Sigma-Aldrich) was dissolved in acetone (15 mL) and methanol (5 mL). The different steps of the process were carried out as reported in [16]. Finally, sensors were annealed for 4 h in synthetic air at 350 °C. The temperatures used are guaranteed not to damage the Kapton^®^ substrate, according to the specifications [17]. Processed substrates were subjected to inspection via optical and scanning electron microscopy, which confirmed that they had not suffered shrinkage, wrinkling, or structural damage.

#### 2.1.2. Silver Stenciled Transducers with Palladium Nanoparticle-Decorated WO_3_ NWs Active Layer

To fabricate these sensors, the procedure followed was the same as that described above (see Section 2.1.1). However, 1.5 mg of palladium (II) acetylacetonate (Pd(C_5_H_7_O_2_)_2_ Sigma-Aldrich), in addition to the 50 mg of tungsten hexacarbonyl, was dissolved in acetone (15 mL) and methanol (5 mL) to obtain Pd-decorated WO_3_ nanowires in a single-step AA-CVD process. This methodology had been employed previously to achieve tungsten oxide nanowires decorated with different noble metals such as Pd [18], Au [12,19], Pt [12,14,20], or Ir [21]. The AA-CVD growth conditions for achieving any of these noble metal-loaded WO_3_ NWs (solvents, temperature, and growth duration) are very similar and compatible with the direct growth of such nanomaterials onto flexible polymeric substrates. Therefore, here Pd-WO_3_ NWs have been chosen as an example case to illustrate the performance of noble metal-decorated tungsten oxide on flexible substrates.

#### 2.1.3. Gold Inkjet-Printed Transducers with Palladium Nanoparticles-Decorated WO_3_ NWs Active Layer

Inkjet-printed electrodes were designed with a size of 18 mm × 15 mm and a sensing area of 4 mm × 2 mm. Track width was set to 380 µm, and the inter-electrode gap was set to 320 µm. Electrode and heater patterns were printed using gold ink (NPG-J, Harima Chemicals Inc., Tokyo, Japan). Firstly, substrates were cleaned following the aforementioned process (see Section 2.1.1). After that, prior to the printing step, the Kapton foil was subjected to an oxygen plasma treatment at 20 W for 2 min with the aim of increasing its hydrophility. Then, the tracks were printed using a Dimatix DMP-2850 materials printer equipped with a DMC-11610 cartridge (Fujifilm Dimatix Inc., Santa Clara, CA, USA) and subsequently annealed at 250 °C for 3 h. Lastly, an active layer of Pd-decorated WO_3_ nanowires was deposited as described in Section 2.1.2.

### 2.2. Gas Sensing Tests

The active layer used in these sensors was employed as a chemoresistive material. Therefore, the response to gases was measured as a change in the electrical resistance of the devices. In this paper, gas sensing tests consisted of measuring the DC current through the sensing electrode set at a constant voltage (Vsens). For this purpose, a power supply (E3631A, Agilent, Santa Clara, CA, USA) was used to apply the required voltages to the electrode (Vsens) and the heater (Vheater), being Vsens > Vheater. Throughout the paper, sensor response is defined as Rair/Rgas-1, where Rair is the baseline sensor resistance in clean air and Rgas is the steady-state sensor resistance under a given concentration of hydrogen. The objective of the test was to compare the sensor performance before and after repeated bending, not to measure peak performance. Therefore, for each sensing material, the working temperature was adjusted to the minimum known suitable for measuring H_2_ [18], in order to save energy and avoid adding extra mechanical stress to the one produced by the flexion, which was the object of study. That is, the operating temperature was set to 150 °C for pristine NW sensors and 100 °C for Pd-decorated NW sensors. These temperatures were set by a computer-based close-loop control system that continuously applied a voltage to the heater and measured the current through it. The control law was deduced from the relationship Rh = R0 (1+α·T), where Rh is the heater resistance, R0 is its resistance at 0 °C, α is the temperature coefficient in °C^−1^, and T is the operating temperature. These parameters were obtained via a previous characterization of the heaters. 

Gas detection was carried out in a Teflon chamber (20 cm^3^) under a continuous gas flow of 100 sccm. The gas concentrations and flow rate were controlled through two PC-driven mass flow controllers (Bronkhost Hitech 7.03.241, Ruurlo, The Netherlands). Thus, sensors were kept under a flow of dry air, and pulses of different H_2_ concentrations were introduced regularly. These concentration pulses caused current variations, which were measured before and after each sensor was subjected to repeated bending cycles.

### 2.3. Bending Cycles

**Figure 2:**
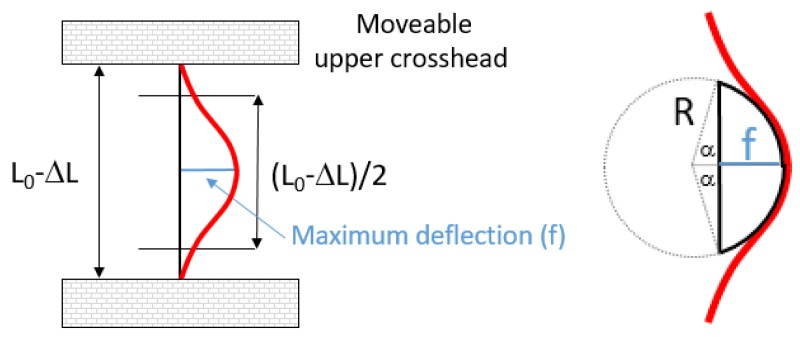
Schematic representation of the experimental setup of the buckled sample where the main parameters are highlighted (**right**), relationship between radius of curvature and deflection (**left**).

To analyze the behavior of a flexible sensor under bending, a compression force can be applied along its longitudinal axis, producing an out-of-plane bending due to buckling. The sample is consequently deformed into a curve, which is called the deflection curve of the sensor. In order to measure this bending behavior in a reproducible and reliable way, flexible sensors can be bent cyclically with an electromechanical universal testing machine. In this case, the flexible sensor is gripped by its ends, placing only the electrode and the active layer area between the grips. Then, a vertical displacement is imposed cyclically to produce the compression force. Depending on the initial distance between the grips (L0) and the stroke ΔL (the vertical displacement of the upper crosshead of the testing machine), the maximum deflection (f) and the approximated radius of curvature can be computed according to Figure 2 and Equation (1). The deflection is computed assuming that both ends are fixed, the buckled length of the sample is (L0-ΔL)/2, and the shape adopted on buckling is parabolic. Integrating along the length of the buckled sample, Equation (1) can be deduced [22].

(1) $$\frac{8\left|f\right|\sqrt{{\left(L_{0}-\Delta L\right)}^{4}+64f^{2}{\left(L_{0}-\Delta L\right)}^{2}}+\sinh^{-1}\left(\frac{8\left|f\right|}{\left(L_{0}-\Delta L\right)}\right)l^{3}}{64\left|f\right|\left(L_{0}-\Delta L\right)}=\frac{L_{0}}{4}$$

**Figure 3:**
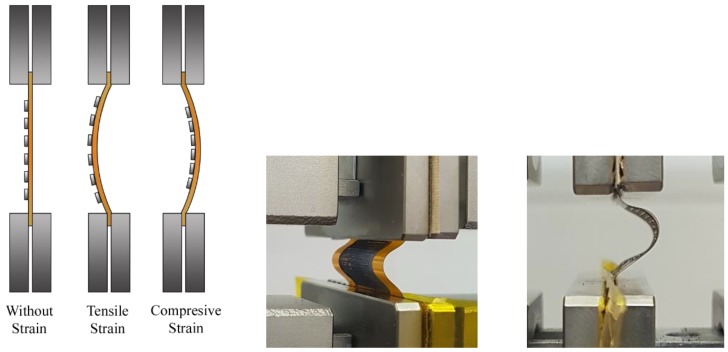
Scheme of the sensor in different bending conditions (**left**). Images of a sensor under tensile strain (**center**) and under compressive strain (**right**).

For the bending test, an AGS-X 10 kN Shimadzu universal testing machine was used. The sample was carefully gripped by its ends to avoid damaging it and ensuring that it remained perfectly aligned with the center of the grips. The machine was programmed in compression mode, i.e., the upper crosshead went down a controlled vertical displacement, or stroke (ΔL), and returned up to complete a cycle. During this cycle, the compression force was exerted, and the sample buckled achieving a maximum deflection (f). The parameters measured in this test were the electrical resistance, the stroke (ΔL), and the force applied (F). Trapezium X Materials Testing software was used to acquire the stroke and force parameters. Simultaneously, an external data acquisition switch unit (34972A Keysight, Santa Rosa, CA, USA) was used to measure the instantaneous electrical resistance of the active layer. One sensor was tested at a time. Depending on the shape of the buckling deflection, the active electrode would be subjected to tensile strain (and stress) or to compressive strain (and stress). Figure 3 depicts the sensor in flat mode under tensile strain and compressive strain.

Sensors under test were subjected to 50 bending cycles at 20 mm/min in tensile strain. In order to apply a comparable mechanical stress, different strokes were applied to devices of different dimensions. The stroke applied to sensors with an Ag-stenciled electrode and the heater was 4 mm, which caused a maximum deflection (f) of 4.72 mm and a radius of curvature of 3.72 mm. The stroke applied to sensors with Au inkjet-printed electrodes was 2 mm, which caused a maximum deflection (f) between 3.18 and 3.23 mm and a radius of curvature between 3.35 and 3.46 mm.

It is worth noting that the loading speed of the crosshead displacement imposed is relatively high in comparison to the values generally reported for this type of test. A 20 mm/min speed was selected in an attempt to approach the flexion to which a wearable would be subject to on a daily use application. Therefore, the samples were subjected to more demanding bending tests (i.e., prone to cause more damages) than those in which the same bending had been applied more progressively. Moreover, curvature radii lower than 4 mm represent very harsh conditions that are well beyond what a wearable can be expected to suffer. 

## 3. Results

**Figure 4:**
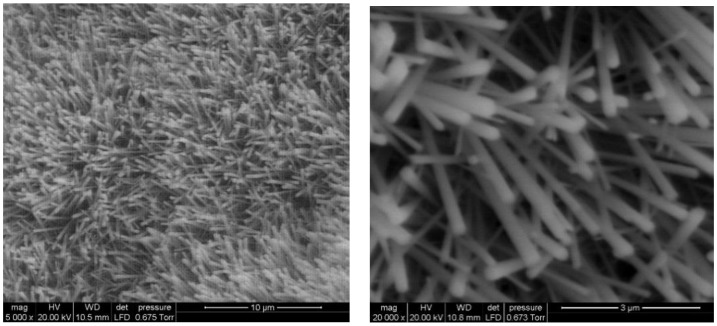

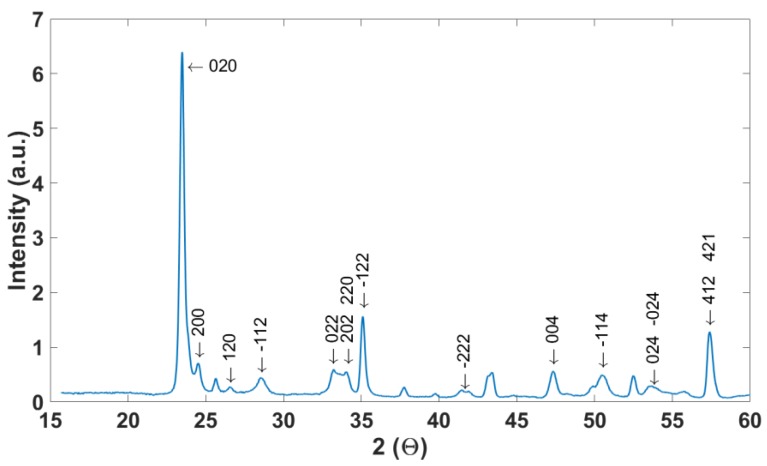
E-SEM images (**upper** panel) and XRD pattern (**lower** panel) of a typical gas sensitive layer consisting of randomly oriented WO_3_ nanowires (NWs).

Prior to the sensing tests and bending cycles, a sample of each set of sensors was inspected to validate the growth of WO_3_ NWs. An environmental scanning electron microscope (E-SEM, FEI QUANTA600, Hillsboro, OR, USA) was employed to examine the structure of the WO_3_ NWs. Images revealed a layer of non-aligned nanowires (Figure 4). Pristine and Pd-decorated WO_3_ NW were successfully grown over the sensing area, connecting electrodes and heaters. Tungsten oxide NW oxidation was incomplete due to the low annealing temperature used to avoid damaging the polymeric substrate. More complete oxidation has been reported at higher temperatures [16]. XRD reveals that both pristine and Pd-loaded tungsten oxide are monoclinic, which is in full agreement with previous results achieved using this growth technique on rigid substrates [16,18].

The following subsections show the results of H_2_ sensing tests for each type of devices before and after undergoing the bending cycles.

### 3.1. Pristine WO_3_ NW over Ag-Stenciled Transducers

**Figure 5:**
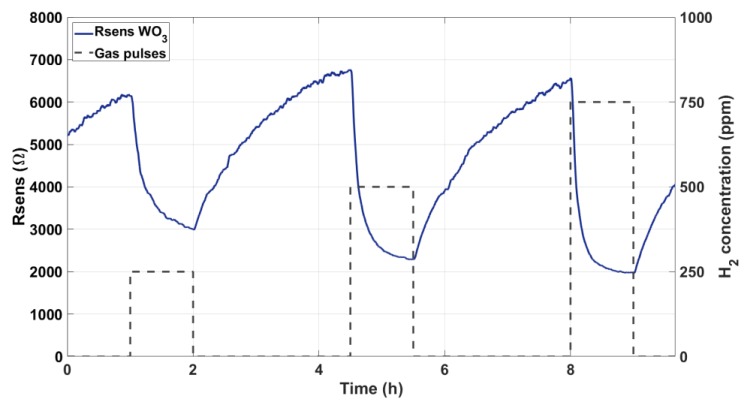
Variation of the sensing layer resistance in response to different H_2_ concentrations.

Sensors with pristine WO_3_ NWs over Ag electrodes were subjected to gas tests for two days. As described above, a continuous flow of synthetic dry air was kept, and different concentrations of H_2_ (250, 500, and 750 ppm) were repeatedly injected. Figure 5 shows a typical measurement of the variation suffered by the resistance (Rsens, i.e., applied Vsens divided by measured Isens) of one of such devices for a complete exposure and recovery cycle of three hydrogen concentrations.

**Figure 6:**
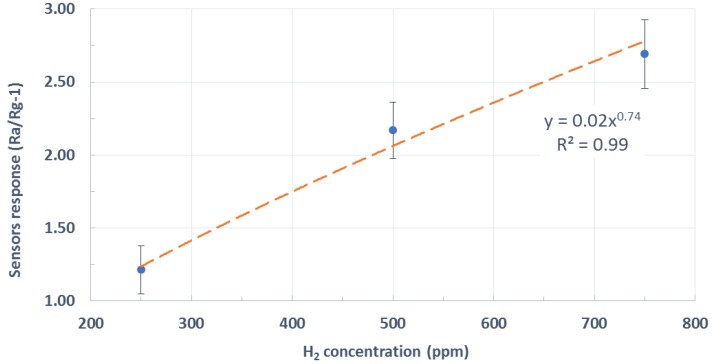
Average response (Rair/Rgas-1) toward hydrogen for three different concentrations. Rair: the baseline sensor resistance in clean air, Rgas: the steady-state sensor resistance under a given concentration of hydrogen.

Figure 6 summarizes the results by showing a calibration curve averaged over the whole set of measurements performed. This included using pristine WO_3_ NWs over Ag-stenciled transducers from different fabrication batches. Error bars represent the standard deviation associated to the measurements of a given concentration. This uncertainty in the measurements is caused by the electrical noise, drift, and differences among fabrication batches. The red dashed line is an interpolation of sensor response employing a potential model, which is typically used in metal oxide gas sensors [23]. 

**Figure 7:**
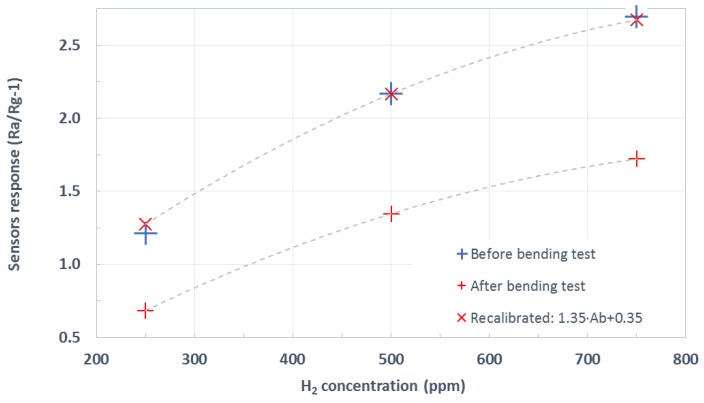
Pristine WO_3_ sensor response before and after completing the bending tests.

**Figure A1:**
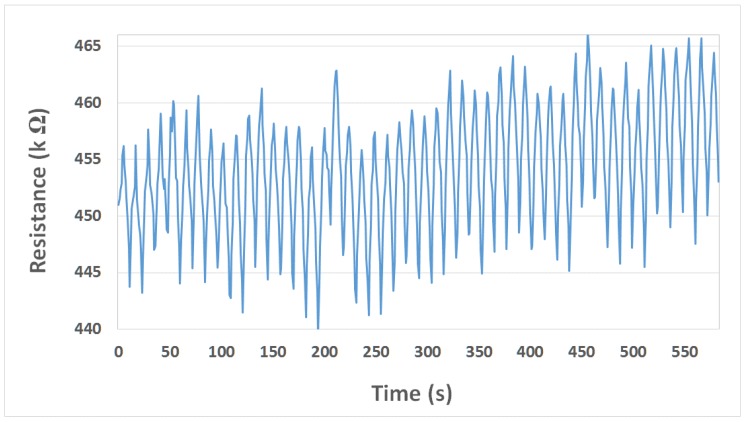
Pristine WO_3_ over stenciled Ag sensor continuous resistance measurement while bending.

**Figure A2:**
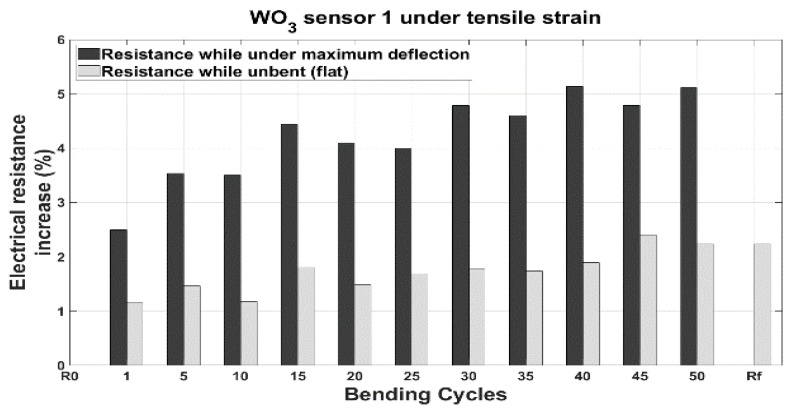
Pristine WO_3_ over stenciled Ag sensors resistance relative increments after bending.

Sensors were bent cyclically 50 times to a curvature radius of 3.7 mm. The electrical resistance of the active layer was continuously measured while bending. It increased and decreased accordingly to the stroke value applied, as expected (see Figure A1). From this figure, it is clear that the resistance of the active layer suffered a small increment after each bending cycle. Figure A2 summarizes such accumulated resistance increase. At the end of the tests, the electrical resistance of the active layers almost recovered their starting value (the increase in the electrical resistance was lower than 2.3%). An optical inspection showed no appreciable damage to the devices. Both facts indicate that these devices had withstood the mechanical stress. Therefore, they would remain operational after repeated bending. Figure 7 summarizes the results of hydrogen measurement after the bending tests. This figure compares the previously calculated calibration curve (shown in Figure 6) to the resulting one after bending. As can be observed, the shape remains almost identical, up to the point that sensor response can be recalibrated by means of a simple linear adjustment to fit the pre-bending response. The sensitivity (i.e., the slope of the calibration curves shown in Figure 7) toward the hydrogen of pristine WO_3_ NWs over Ag-stenciled transducers showed a reduction by a factor of 1.35 after the bending exercise.

### 3.2. Pd-Decorated WO_3_ NW over Ag-Stenciled Electrodes

**Figure 8:**
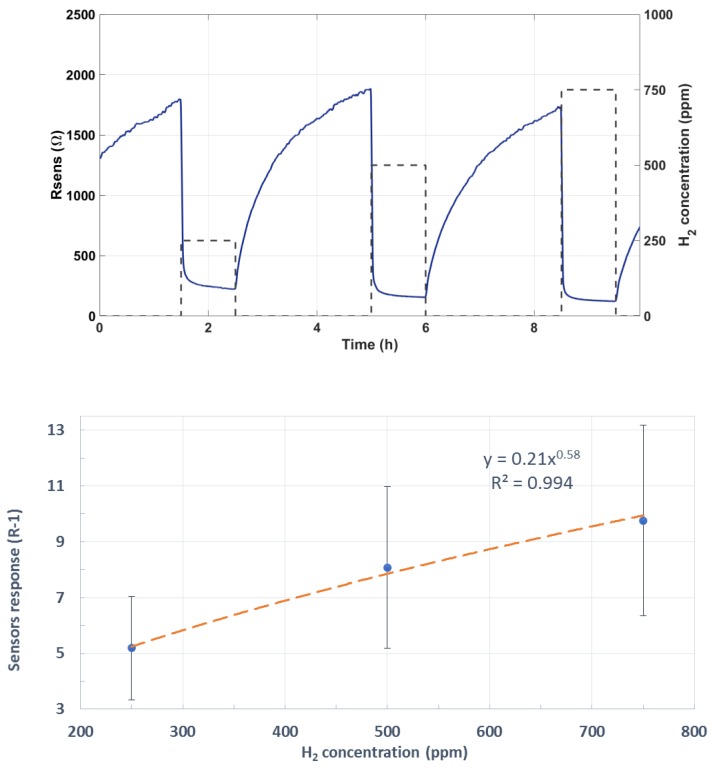
Resistance response to H_2_ (**upper** panel) and average calibration curve for Pd-decorated sensors (**lower** panel).

The same procedure was followed with Pd-decorated NW sensors. Figure 8 shows in its upper panel a typical measurement of the variation suffered by the resistance of one of such devices for a complete exposure and recovery cycle to three hydrogen concentrations. The lower panel in Figure 8 shows an averaged calibration curve.

The responses of Pd-loaded tungsten oxide sensors were significantly higher than those of pure tungsten oxide. This can be attributed to the well-known catalytic activity of palladium nanoparticles (NPs) for enhancing H_2_ sensing. It has been demonstrated that palladium NPs modify the Fermi level of the supporting semiconductor material (here tungsten oxide) and hence its conductance, which is in accordance with previous works with Pd–WO_3_ nanowires over rigid substrates [18]. The response mechanism is further described below in Section 4. The response times (t_90_) toward hydrogen of bare and Pd-loaded WO_3_ NW sensors are reasonably similar to the state of the art. Namely, t_90_ are a few minutes and below one minute for bare and Pd-loaded tungsten oxide sensors, respectively. In contrast, recovery times are rather long. The reason for this is twofold. On the one hand, sensors were operated at quite low operating temperatures (up to 150 °C), and this is always detrimental for sensor dynamics. On the other hand, given the experimental design implemented, sensors were exposed to hydrogen pulses that lasted very long (1 h), which makes the process of cleaning the sensor surface longer.

**Figure 9:**
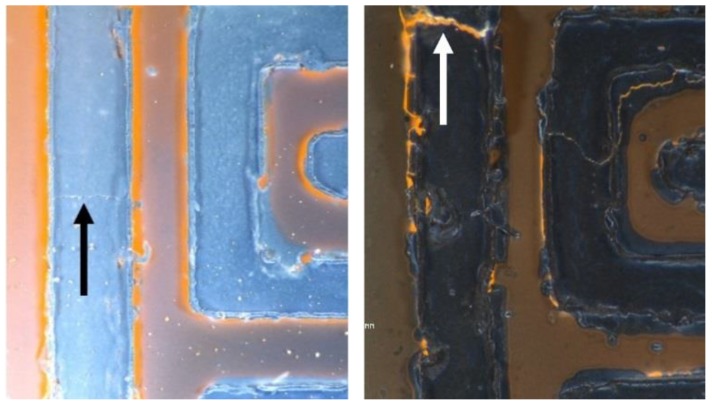
Optical images of two sensors employing stenciled silver inks after the bending and gas sensing tests. Left: pristine WO_3_ NW sensors Right: Pd-loaded WO3 NW sensor. Arrows indicate the onset of a fracture (**left**) or the occurrence of a fracture with delamination (**right**).

**Figure A3:**
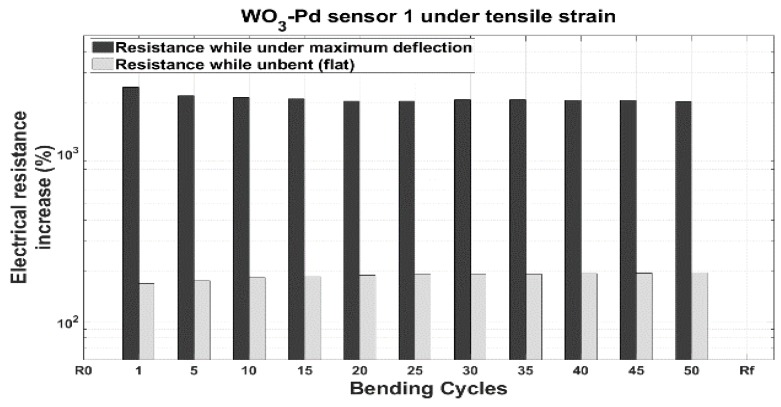
Pd-decorated WO_3_ over stenciled Ag sensors resistance relative increments after bending.

Sensors were bent cyclically 50 times to a curvature radius of 3.4 mm. While bending, the electrical resistance of the active layer increased and decreased according to the stroke value, as expected (Figure A3). However, during this test, the delamination of conductive tracks was observed, and the measured resistance values increased over 1000 %. At the end of the tests, the electrical resistance of the devices had increased permanently, foretelling the occurrence of structural damages. An optical inspection of bent substrates showed the presence of significant cracks due to delamination. These cracks compromise electrode continuity. Figure 9 shows how different the state of conductive tracks is after the gas sensing and bending tests in pristine and Pd-decorated sensors.

For the pristine WO_3_ sensor, we can distinguish a slight line where a fracture could start (black arrow points at this line). For the WO_3_-Pd sensor, we can observe noticeable track fractures and detachments/delamination (white arrow points at one of these fractures). As expected from the dramatic, irreversible changes observed in baseline resistance after the bending test, the Ag tracks were so badly damaged that no noticable sensing results were obtained. Hence, no calibration curve for this type of sensor after their bending test is provided. It is concluded that the presence of Pd during the direct growth of Pd-loaded tungsten oxide NWs over polymeric substrates having Ag-stenciled tracks leads to catalytic reactions that fragilize these Ag tracks.

### 3.3. Pd-Decorated WO_3_ NWs over Au Inkjet-Printed Electrodes

**Figure 10:**
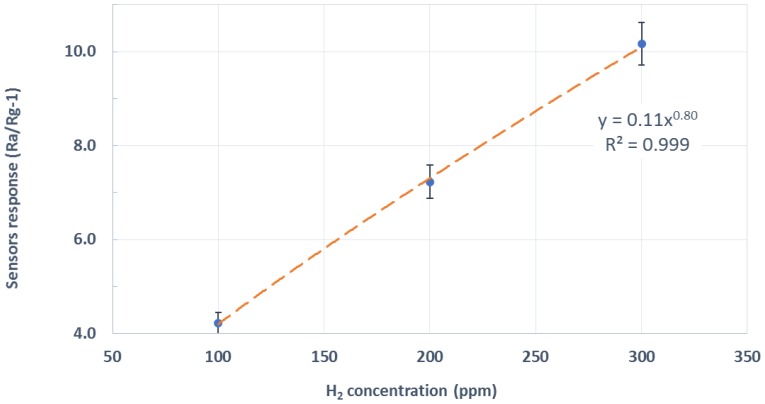
Average response (Rair/Rgas-1) toward different hydrogen concentrations of Pd-decorated WO_3_ sensors that employed gold inkjet-printed electrodes.

To overcome the fragility of the Ag tracks affected by the catalytic behavior of Pd nanoparticles, gold inkjet printed electrodes were produced (as explained in Section 2.1.3). The usual testing procedure was employed with these sensors. Figure 10 shows the resulting averaged calibration curve of these devices prior to bending. It is worth noting that this is a more expensive fabrication method, given the cost of the Au ink. On the other hand, these devices were significantly more reproducible than the stenciled ones. Hence, the standard deviation was dramatically reduced. As already observed, the response of Pd-loaded sensors is several times higher than that of pristine WO_3_ sensors.

**Figure A4:**
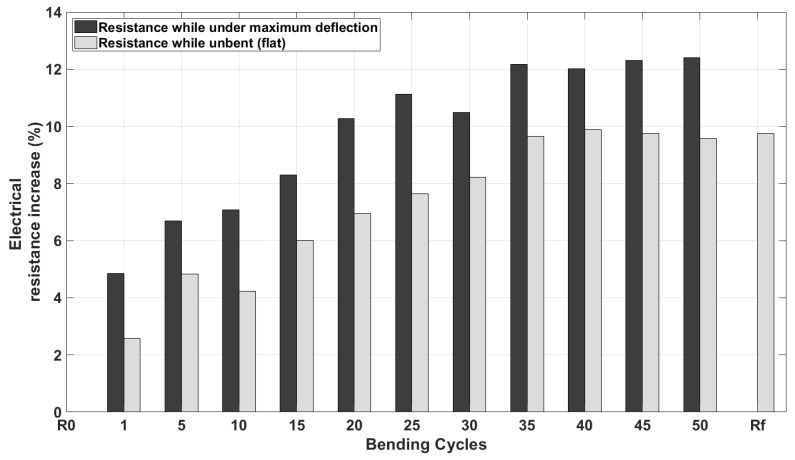
Pd-decorated WO_3_ over inkjet-printed Au sensors resistance relative increments after bending.

The inkjet-printed sensors were cyclically bent 50 times, and the maximum deflection (f) due to buckling was 1.47 mm, producing a radius of curvature of approximately 3.4 mm. During the tests, there were no detachments/delamination (active layer or tracks). The electrical resistance increase while the sensor was under maximum deflection was about 12%, and the permanent resistance increase was about 10%, which was significantly lower than those recorded for stenciled sensors (Figure A4). These results indicate the absence of damages after the bending test, and therefore, that these sensors remain fully functional after the bending test.

**Figure 11:**
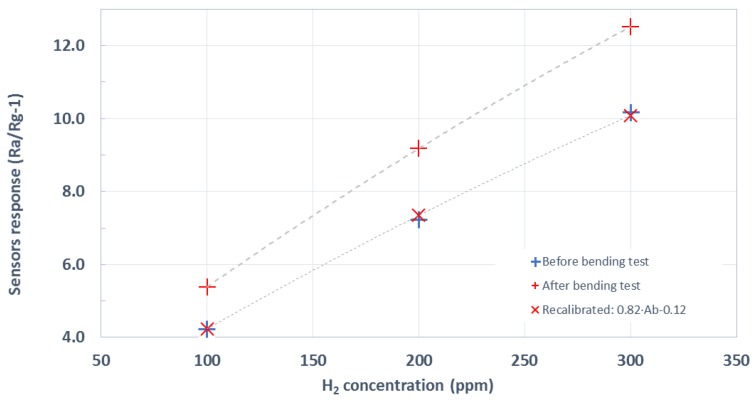
Hydrogen response of Pd-decorated WO_3_ over inkjet-printed substrates before and after having undergone bending tests.

Figure 11 shows the results of the gas tests conducted after bending, comparing the previously calculated calibration curve to the resulting one after bending. As can be observed, the response has increased after bending, but the shape remains basically identical and can be recalibrated by means of a simple linear adjustment to fit the pre-bending response. In this case, it can be concluded that the sensitivity (i.e., slope of the calibration curves shown in Figure 11) of Pd-loaded tungsten oxide NW sensors showed an increase by a factor of 1.22 after the bending, once the delamination problem had been solved by replacing the stenciled Ag tracks by Au inkjet-printed ones.

## 4. Discussion

Three different sets of MOX gas sensors were fabricated. Each one was tested against H_2_, cyclically bent, and then tested against H_2_ once more to determine the effects of bending in sensing performance. 

### 4.1. Hydrogen Detection Mechanism and Moisture Cross-Sensitivity

At the operating temperatures employed here, Pd nanoparticles increased the number of oxygen adsorbed species at the surface of tungsten oxide (in comparison to bare WO_3_), favoring the dissociation of the H_2_ molecule-forming H^+^ species, and via a spillover effect, their diffusion onto the surface of WO_3_ NWs where they react with oxygen adsorbates [18,24]. This explains the superior performance of Pd–WO_3_ in the detection of hydrogen.

The effect of ambient moisture in the response toward hydrogen of either pure or Pd-loaded WO_3_ NWs synthesized via AA-CVD has been reported previously [18]. When ambient conditions are changed from dry to 50% R.H., the baseline resistance of pure WO_3_ NWs experiences a 50% decrease, and the response toward hydrogen decreases by about 30%. In contrast, Pd-loaded WO_3_ NWs suffer only a slight change in their baseline while the hydrogen response remains almost unaltered.

### 4.2. Discussion on the Results of the Bending Tests

It has been reported that the most common failures for brittle films on flexible substrates are film cracking, channeling, and delamination [25]. Cracks initiate and grow during the bending process to reduce stress. Cracks commonly generate in the functional coatings long before the substrate fails and tend to originate around sites related to surface defects [26]. Since the substrate used in this work is an inexpensive commercially available polymer, it is very likely to have a significant amount of defective areas that could originate cracks under bending. Cracks generate permanent changes in the electrical resistance of the active layer, and therefore modify the sensor baseline. In addition, during the bending test, NWs may change orientation and their NW-to-NW distance, which possibly modifies the binding energy and charge transfer between the gas-sensing layer and the target gas molecules, producing slight variations (positive or negative) in sensor response [27]. Therefore, despite we have obtained significantly different results for slight changes in the fabrication procedure, all these results are in agreement with what has been reported in the literature. This is a relevant result, taking into account that the devices tested were subject to an out-of-plane deformation due to buckling. A buckling deformation process is considered an unstable elastic phenomenon that produces a critical stress state that collapses the element in an undesirable dynamic mode [28,29]. Besides, the cyclic loading imposed (strain level, speed, and cadence) may well be associated with functional fatigue, i.e. a worsening effect in the main functional properties with an increasing cycle numbers. This mode of fatigue, unlike traditional structural fatigue (with higher number of cycles), does not lead to the failure of the device but could significantly limit its service life. Thus, analyzing the mechanical behavior of a flexible device under buckling can be considered the harshest bending mode, since it imposes the highest deformation speed. Hence, in comparison to most reports (Table A1), our bending tests were harsher considering curvature radius and bending speed. 

## 5. Conclusions

From this process and its results, there are many conclusions to be drawn. Firstly, we have shown that it is possible to produce reliable, flexible gas sensors with simple and affordable technologies. Stenciling Ag electrodes is an inexpensive and quick prototyping technique, but it is not highly reproducible and is prone to degradation. Au electrodes are far more stable but also more expensive. Yet, as inkjet printing is an additive process, it remains affordable in comparison to most clean-room photolithographic processes. Both stencil and inkjet printing have been used to produce WO_3_ chemoresistive sensors that can withstand harsh bending conditions and afterwards remain fully functional for gas sensing. Moreover, in both cases, the response change after bending can be compensated for via a linear adjustment that can be easily implemented by the data acquisition software: a constant offset equal to the response variation of the sensor while in air and a gain factor equal to this constant plus one. Keeping in mind that changes in resistance due to flexure are three orders of magnitude faster than those due to changes in analyte concentration, it should be easy for the control software to compensate them and correct the calibration curve in real time.

Secondly, not every working sensor fabricated over a flexible substrate is actually a flexible sensor. The sensors that used Pd-decorated WO_3_-NWs over Ag-stenciled tracks were fabricated following equivalent procedures to those used for pristine WO_3_ NW sensors, and they worked properly before the bending tests. However, the slight change in the AA-CVD precursor composition (i.e., the inclusion of the Pd precursor to the mixture) made the electrodes brittle, which in turn made the full system prone to failure. That leads to the next two conclusions.

**Figure A5:**
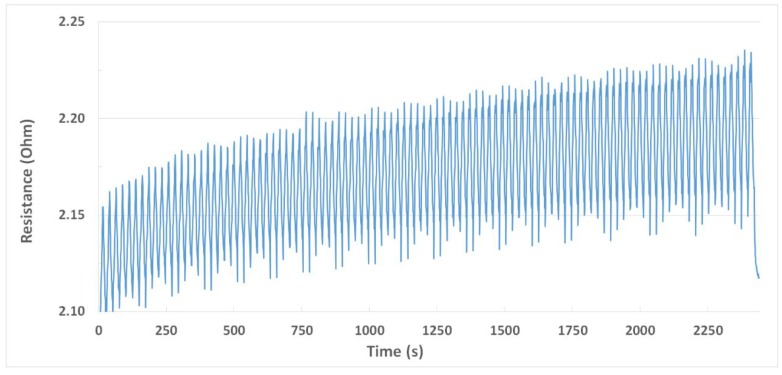

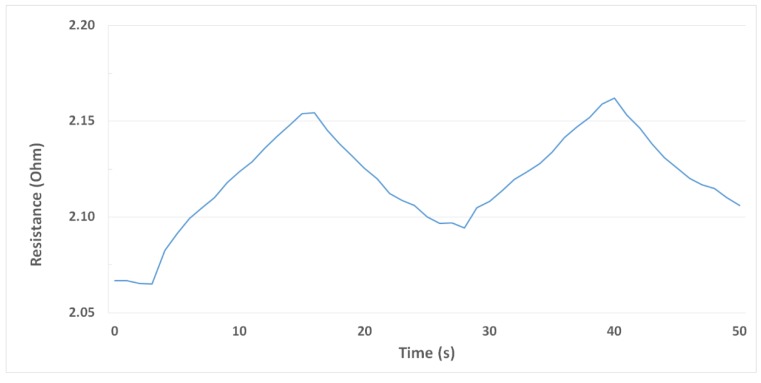
Stenciled Ag track continuous resistance measurement while bending (**upper** panel). Stenciled Ag track continuous resistance measure while bending (zoom in) (**lower** panel).

**Figure A6:**
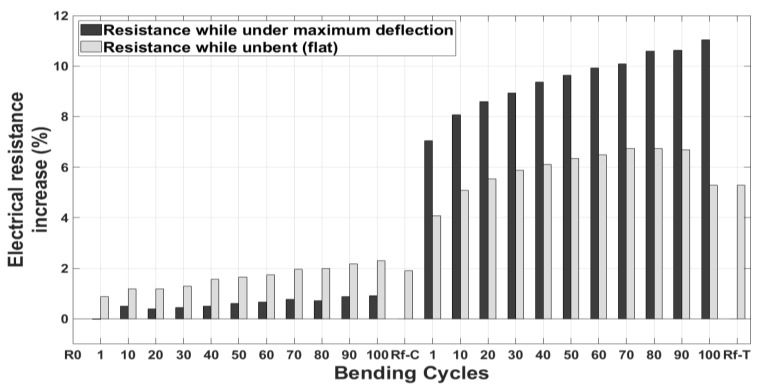
Evolution of the relative increment in electrical resistance for stenciled Ag tracks subject to 100 bending cycles under contraction followed by 100 bending cycles under traction.

Thirdly, metallic tracks play a significant role in the complete system flexibility. Bare Ag-stenciled electrodes showed good resilience to bending tests (see Figure A5 and Figure A6). Films are expected to suffer from cracks when subjected to compressive stress [25,30]. In this case, only microcracks appeared that increased the heater resistance from 2 Ω to 2.1 Ω when flat and to 2.2 Ω when buckled, even after having undergone 100 cycles of compression plus 100 cycles of traction bending. Even though it could seem that a few Ohm resistance in series with many thousand Ohms from the active layer would not make a noticeable difference, at the end of the whole fabrication process, metallic tracks can result in the weak link in the chain. Their chemical interaction with the active layer can lead to a fragile 3D structure, even starting as a working system but evolving to a failure while in field operated.

Lastly, a standard procedure should be defined to assess the flexibility of sensors. On the one hand, nowadays, it is difficult to compare academic results if there is not even a de facto standard. It seems that, at least, the curvature radius (more representative than curvature angle as devices may have very different dimensions), loading speed and number of repetitions should be agreed upon. On the other hand, such a standard should be related to sensor application. A sensor conformed to a column for structural health monitoring in buildings will not be as stressed as a wearable attached to a sportswear fabric. So, in the future, ideally, one should be provided with different specification tables to be met so that a device may qualify as flex-electronics in order to be used in one or another particular application.

## Appendix A

**Table A1:** Gas sensors based on metal oxide (MOX) NW/NR active layers over flexible substrates. NR: nanorods.

Sensing Layer	Bending Conditions	Performance Comparison	Observations	Reference
Si NWs	Not Bent	No	-	[10]
WO_3_ NWs	Not Bent	No	-	[31]
ZnO NWs	Not Bent	No	-	[32]
ZnO NRs	Not Bent	No	-	[33]
Pd@Ag HNWs	Angle = 30° Radius = 4 mm	Yes	No Clear Statement of Bending Speed and Cycle Timing. Base Resistance and Sensing Characteristics Such as Sensing Speed Look Relatively Unstable During the H_2_ Sensing Reaction.	[11]
WO_3_–Au /WO_3_–Pt	(a) Angle < 45° (b) Angle > 45°	No	Unknown Bending Speed, Angle Measured Including Terminals (So Actually Less Stress Applied to Sensing Area). (a) Reversible Increase of the Sensing Film Resistance. (b) Detachment of the Layer that Forms the Contacts after Bending.	[12]
ZnO NRs-Gr/M	Radius <8 mm 100 cycles	No	No Clear Comparison of Performance before and after Bending Tests. Unknown Bending Speed; Angle Measured Including Terminals (So Actually Less Stress Applied to Sensing Area)	[34]
Pd-decorated ZnO NRs	Angle: 0°, 45°, 90°	Yes	Bent 10^6^ cycles at 90°, Response Stability until 10^5^ cycles. No Significant Degradation of Performance was Observed Prior to 10^6^ cycles. Unknown Bending Speed, Angle Measured Including Terminals (So Actually Less Stress Applied to Sensing Area).	[35]
Pd NP-coated ZnO NWs	Radius: 7.5 mm, 10 mm Cylindrical support	Yes	Bent over a Cylindrical Object, which Ensures a Smooth Curvature and Avoids Excessive Stress Around Defective Surface Areas. Low Bending Speed. Slight Changes of Sensing Performance were Observed by Mechanical Bending.	[36]
Pt-Pd/ZnO NRs	Angles: 0°, 20°, 40°, 60°, 90° Cylindrical support 106 cycles	Yes	Bent over a Cylindrical Object. Stable Response for 105 cycles. Sensing Test while Bent.	[37]
Ag-loaded ZnO NRs	Angles: 0°, 45°, and 90° U-shaped support 5 x 105 cycles	Yes	Stable Response for 5·104 cycles; after that, It Dropped by 8%.	[27]
ZnO NP film	Radius: 6 mm, 2.5 mm Cylindrical support	Yes	Bent over a Cylindrical Object. Small Variation at 6 mm. Cracks when Contracting at 2.5-mm Sensing Test while Bent.	[30]
